# Antibacterial mechanism of chelerythrine isolated from root of *Toddalia asiatica* (Linn) Lam

**DOI:** 10.1186/s12906-018-2317-3

**Published:** 2018-09-26

**Authors:** Nan He, Peiqing Wang, Pengyu Wang, Changyang Ma, Wenyi Kang

**Affiliations:** 1 Institute of Chinese Materia Medica, Henan University, Kaifeng, 475004 Henan China; 2 Kaifeng Key Laboratory of Functional Components in Health Food, Kaifeng, 475004 Henan China

**Keywords:** Chelerythrine, Antibacterial mechanism, *Toddalia asiatica* (Linn) Lam., HPLC, *Staphylococcus aureus*

## Abstract

**Background:**

Antimicrobial resistance was one of serious worldwide problems confused many researchers. To solve this problem, we explored the antibacterial effect of chelerythrine, a natural compound from traditional Chinese medicine and studied its action.

**Methods:**

The contents of chelerythrine from different fractions of *Toddalia asiatica* (Linn) Lam (*T. asiatica*) were determined. The anti-bacterial activities of chelerythrine were tested by disc diffusion method (K-B method). Scanning electron microscopy (SEM), alkaline phosphatase (AKP), bacterial extracellular protein leakage and SDS-PAGE analysis were also used to investigate the antibacterial mechanism of chelerythrine.

**Results:**

Analytic results of High Performance Liquid Chromatography showed that the content of chelerythrine (1.97 mg/g) in the ethyl acetate fraction was the highest, followed by those of methanol fraction and petroleum ether fraction. The in vitro anti-bacterial mechanisms of chelerythrine from *T. asiatica* were assessed. Chelerythrine showed strong antibacterial activities against Gram-positive bacteria, *Staphylococcus aureus* (SA), Methicillin-resistant *S. aureus* (MRSA), and extended spectrum *β*-lactamase *S. aureus* (ESBLs-SA). The minimum inhibitory concentrations (MICs) of chelerythrine on three bacteria were all 0.156 mg/mL. Furthermore, results suggested that the primary anti-bacterial mechanism of chelerythrine may be attributed to its destruction of the channels across the bacterial cell membranes, causing protein leakage to the outside of the cell, and to its inhibition on protein biosynthesis. Images of scanning electron microscope revealed severe morphological changes in chelerythrine-treated bacteria except control, damage of parts of the cell wall and cell membrane as well as the leakage of some substances.

**Conclusions:**

Chelerythrine isolated from root of *Toddalia asiatica * (Linn) Lam possesses antibacterial activities through destruction of bacterial cell wall and cell membrance and inhibition of protein biosynthesis.

## Background

With the increasing and widespread use of antibiotics, a large percentage of microorganisms have developed antimicrobial resistance, causing economic loss and even death. Thus, searching new class of effective antimicrobial agents is essential to cope with the continuous emergence of multi-drug-resistance of bacteria, especially resistance to anti-staphylococcal drugs. More and more phytochemicals derived from plants have been considered as a potential source of promising antibacterial agents [1]. Some components extracted from natural plants showed inhibitory effects against *Staphylococcus aureus* (SA) at low dose. However, the underlying antimicrobial action mechanisms of most natural components are currently unknown [2]. It is quite possible that promising natural compounds can be discovered as the new antibiotic drugs [3].

*T. asiatica*, belonging to the family Rutaceae, distributes in the dry areas full of hedges and bushes [4]. The root and bark of *T. asiatica* have been used in traditional medicine to treat malaria, diarrhea, cholera and cough [5]. Its leaves have been used to treat lung and skin diseases, and rheumatism [6]. Moreover, the plant also possesses antimicrobial, larvicidal, antidiabetic, antioxidant, antinocieptive and anti-inflammatory activities [7–9]. It has been reported that the root and duramen of *T. asiatica* are mainly rich in coumarins, triterpenoids and alkaloids [10–12]. In our previous research, we isolated thirteen compounds from the petroleum fraction and the ethyl acetate fraction of roots of *T. asiatica* and identified them [13, 14]. These compounds were screened out based on anti-bacterial activities. Among them, chelerythrine showed more effective and potent antibacterial activity. Chelerythrine is a kind of benzo [c] phenanthridine alkaloids with many pharmacological activities, such as anti-cancer, anti-bacterial, anti-inflammatory, insecticide, anti-fibrosis activities, etc. [15–19]. In past decades, a majority of studies were focused on its anti-cancer and anti-bacterial activities. It has been suggested by a previous study that chelerythrine may possess antibacterial activities and its antibacterial action mechanisms of chelerythrine against bacterium may be related to its inhibitory effects on DNA synthesis, proteinase synthesis and membrane permeability of bacterium [20]. However, its exact action mechanisms against bacteria are currently unclear and need be further elucidated. Therefore, in this paper, we focused on elucidating its antibacterial mechanisms by detecting the changes in cell wall and cell membrane electrical conductivity, alkaline phosphatase (AKP), extracellular proteins, electrophoresis protein bands with SEM and TEM.

## Methods

### Bacterial strains and bacterial culture

*Staphylococcus aureus* (SA) 25,923 was purchased from Shanghai Tiancheng Bio-information and Technology Co., Ltd., (Shanghai, China). MRSA and ESBLs-SA were provided by Huaihe Hospital (Kaifeng, Henan, China), and identified by Vitek-AMS (Automated Microbic System).

The three *S. aureus*s trains were activated and inoculated into Broth Agar Medium at 37 °C for 24 h in a thermostat, and then bacterial concentration was diluted with sterile broth agar to 10^6^ CFU/mL.

### Preparation of extracts and drugs

The roots of *T.  asiatica* (200801) were collected from Guizhou province, China, in September 2008 and identified by Professor Zhiyou Guo, Qian Nan Normal College for Nationalities, Guizhou, China. The voucher specimen was stored at the Institute of Chinese Materia Medica, Henan University (Kaifeng, Henan, China).

Root powder of *T. asiatica* (1.3 kg) was extracted three times with methanol for 7 days each time. Then the extracts were evaporated and dried under reduced pressure. The concentrated extract was mixed with silica gel, and eluted successively with petroleum ether, ethyl acetate and methanol to obtain petroleum ether fraction, ethyl acetate fraction and methanol fraction, respectively.

Ethyl acetate fraction was loaded to silica gel column and eluted with CH_2_Cl_2_: MeOH (v:v = 100:1~ 8:2). Ten sub-fractions were obtained. After the sixth subfraction was repeatly subjected to silica gel column and Sephadex LH-20, chelerythrine (24.5 mg) was obtained. The purity of chelerythrine was higher than 98%. The NMR data of chelerythrine were published on China Pharmacist [14].

### Analysis of chelerythrine by HPLC

The HPLC analysis was carried out in an Agilent 1260. Agilent TC-C_18_ column (250 mm × 4.6 mm, 5 μm) with acetonitrile and water containing 0.4% phosphoric acid (30:70) as mobile phase were used. The column temperature was set at 30 °C. The detection wavelength was at 258 nm, the flow rate was 1.0 mL/min and the injection volume was 10 μL.

### Antibacterial activity

Antibacterial activity of chelerythrine was tested by disc diffusion test. Sample solution was obtained after dissolving chelerythrine (50 μg) with DMSO (1 mL). Filter paper discs of 6 mm diameter were impregnated with 5 μL of sample solution. A disc prepared with corresponding volume of DMSO was used as negative control and that prepared with berberine was used as the positive control. The plates were incubated at 37 °C for 24 h. Antimicrobial activity was evaluated by measuring the diameter of the inhibition zone (IZ) [21].

According to the results of IZ method, bacteriostatic ring was > 8 mm, then its minimum inhibitory concentration (MIC) was determined in triplicate by the tube doubling dilution method, and the test sample was serially obtained a series of concentration. MICs were defined as the lowest sample concentration that exhibited IZ.

### Inhibitory effects on the bacterial cell wall

Chelerythrine at MIC and 3 × MIC were mixed with bacterial suspension (5 × 10^6^ CFU). Sterile water was used as negative control group. All the groups were cultured in incubator at 37 °C and time sampling method (0 h, 0.17 h, 0.33 h, 0.5 h, 1 h, 2 h, 3 h, 4 h, 5 h, 7 h, and 9 h) was adopted. Then the testing objects were centrifuged at 3500 rpm for 10 min, the content of AKP in supernatant was measured by the AKP kit assay with time.

### Determination of soluble protein

Coomassie brilliant blue method was used to determine the concentration of soluble protein in bacterial suspension treated with chelerythrine (MIC and 3 × MIC) and the control groups, respectively. After resting for 10 min, OD values of all groups were measured with spectrophotometer at 595 nm.

### SDS-page

The chelerythrine solution at MIC was added to bacterial suspension and the mixed suspension was cultivated at 37 °C for 24 h. Sterile water was used as negative control. Mixed solution (12 mL) was sampled at 3, 6 and 9 h, respectively. After the samples were diluted and adjusted into the same OD value, the precipitations were obtained by centrifuge, and then 80 μL of sterile water and 20 μL of 5 × SDS sample buffer precipitation were added, mixed thoroughly and boiled in a water bath at 100 °C for 5 min, the solution was centrifuged again and supernatant on standby was collected and used as protein extracts.

The vertical slab gel electrophoresis made from 8% of separating gel and 5% of stacking gel was used to separate proteins. 5 μL of protein maker and 15 μL of each sample were drawn with micro pipette tips. At first, the voltage was set at 60 V and electrophoresis was run, when the bands of samples passed into separating gel, the voltage was adjusted to 120 V and electrophoresis was continuously run until the blue ribbons was one or two centimeters distant from the bottom edge of the gel. The power was turned off and rubber blocks were taken out and stained with Coomassie brilliant blue for 3 h. Thereafter, the stained rubber blocks were de-stained with destainer in a shaking table up to appearing clear bands.

### Scanning electron microscope (SEM) and transmission electron microscope (TEM)

Chelerythrine (1 × MIC) was added with bacteria at the concentration of 10^9^ CFU/mL for a shaking Table (150 r/min) for 1.5 h and 6 h at 37 °C, respectively. Distilled water was used as control. The depositions were collected by centrifugation and fixed with 2.5% glutaraldehyde at 4 °C overnight, and washed three times with 0.1 M phosphate buffer solution (PBS, PH 7.2). The samples were dehydrated in a graded series of alcohols (30, 50, 70, 85, 90 and 100%). Tert-butanol was used to replace the ethanol twice before coating onto the metal foil. Eventually, the cells were dried by freeze-drying apparatus (ALPHA1–4, Christ, Germany), and visualized under a scanning electron microscopy (SEM, JSM-5600LV, JEOL, Japan) and a transmission electron microscopy (TEM, JEM-2010, JEOL, Japan), respectively.

## Results

### Determination of Chelerythrine in *T. asiatica*

**Fig. 1:**
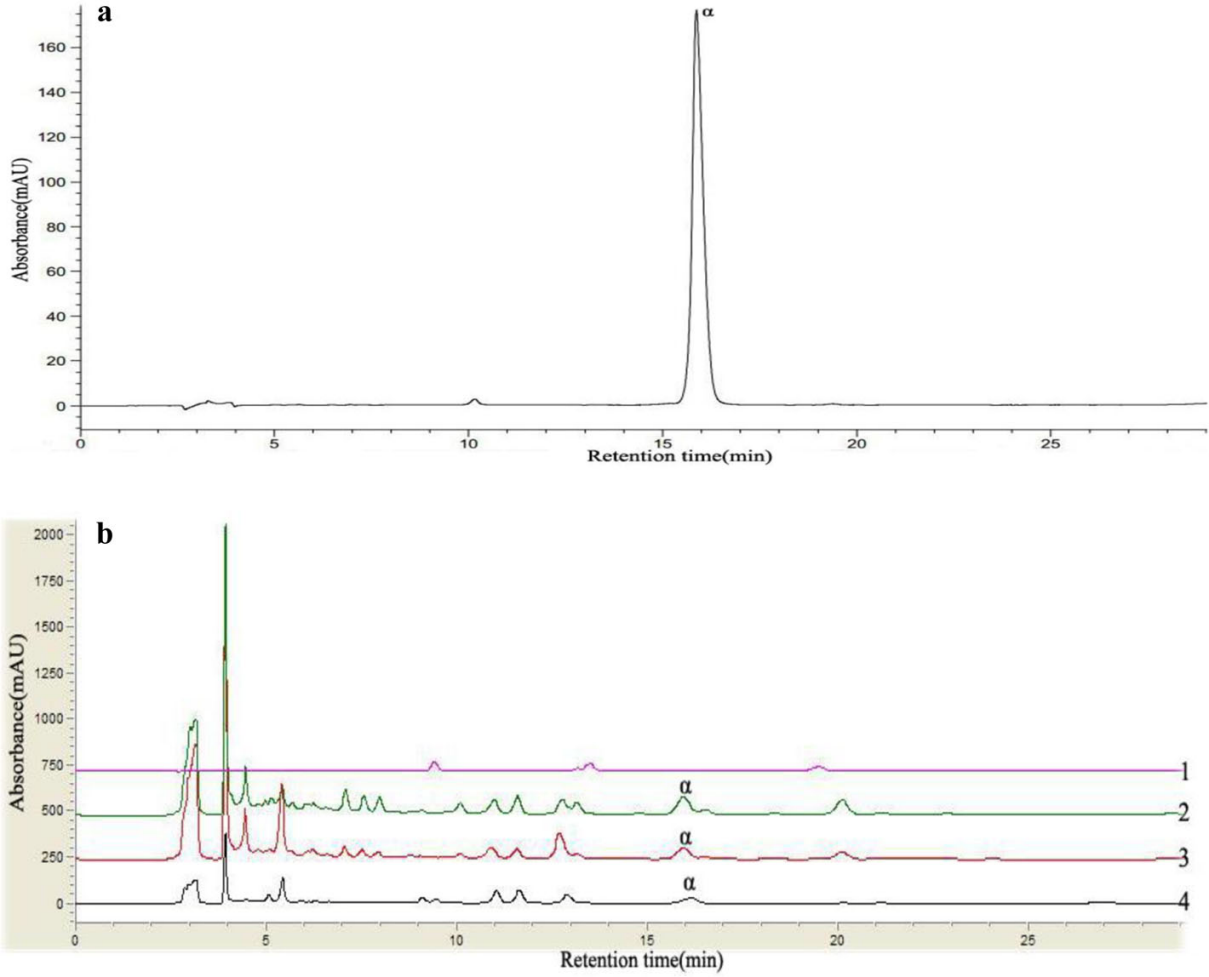
HPLC chromatograms of (**a**) standard and (**b**) *T. asiatica* fractions (peak α, Chelerythrine). Petroleum ether fraction (1), Ethyl acetate fraction (2), Methanol fraction (3), and Methanol extract (4)

The typical chromatograms were showed in Fig. 1. The contents of each sample were presented in Table 1. There content of chelerythrine (1.97 mg/g) in ethyl acetate fraction was the highest among all the samples, while no chelerythrine was detected in petroleum ether fraction.

**Table 1:** Contents (mg/g) of chelerythrine in different fractions extracted from roots of *T. asiatica*

Standard	Petroleum ether fraction	Ethyl acetate fraction	Methanol fraction	Methanol extract
Chelerythrine	ND	1.97	1.19	0.66

*ND* not detected

### Antibacterial activity in vitro

In Table 2 and Table 3, petroleum ether fraction and ethyl acetate fraction of roots of *T. asiatica* inhibited the *S. aureus* and the Methicillin-resistant *S. aureus* at the same concentration (50 mg/mL). The IZs of chelerythrine (10 mg/mL) against SA, MRSA and ESBLs-SA were 19.07 ± 0.19 mm, 18.12 ± 0.14 mm and 16.93 ± 0.23 mm, respectively, and its MICs against three bacteria were 0.156 mg/mL, 0.156 mg/mL and 0.156 mg/mL, respectively.

**Table 2:** Inhibition zones of antimicrobial activity of *T. asiatica* fractions and Chelerythrine

Samples	Concentration(mg/mL)	Inhibition zones(mm)		
		SA	MRSA	ESBLs-SA
Petroleum ether fraction	50	8.13 ± 0.18	9.21 ± 0.18	–
Ethyl acetate fraction	50	8.06 ± 0.13	8.00 ± 0.18	–
Methanol fraction	50	7.95 ± 0.28	–	–
Chelerythrine	10	19.07 ± 0.19	18.12 ± 0.14	16.93 ± 0.23
Blank		–	–	–
DMSO		–	–	–
Berberine	2.5	15.04 ± 0.31	15.89 ± 0.24	16.27 ± 0.19

(−) no activity; Berberine-positive control

**Table 3:** MICs of *T. asiatica* fractions and chelerythrine against SA, MRSA and ESBLs-SA

Tested bacteria	MIC(mg/mL)				
	Pe	Ea	Me	Che	Ber
*Staphylococcus aureus*	50	50	50	0.156	0.0312
Methicillin-resistant *S.aureus*	25	50	NT	0.156	0.0312
Extended spectrum β-lactamases *S.aureus*	NT	NT	NT	0.156	0.0312

*NT* not test, *Pe* Petroleum ether fraction, *Ea* Ethyl acetate fraction, *Me* Methanol fraction, *Che* Chelerythrine, and *Ber* Berberine

### Antibacterial mechanisms of chelerythrine

#### Effects on the cell wall of SA, MRSA and ESBLs-SA

**Fig. 2:**
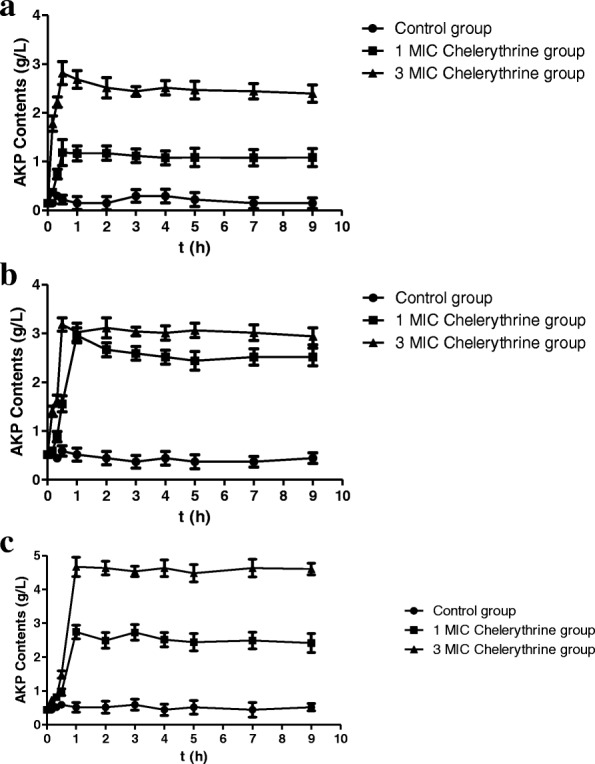
The extracellular activities of AKP against SA (**a**), MRSA (**b**) and ESBLs-SA (**c**)

Fig. 2 showed that after chelerythrine was added to bacteria, the contents of AKP were increased rapidly and reached the highest level in 1 h as compared to those of the control group. There was a positive correlation between the concentration of chelerythrine and AKP leakage, suggesting that chelerythrine can destroy cell wall of bacteria and lead to the increased AKP leakage for a short period of time.

#### Effects on the contents of extracellular proteins of SA, MRSA and ESBLs-SA

**Fig. 3:**
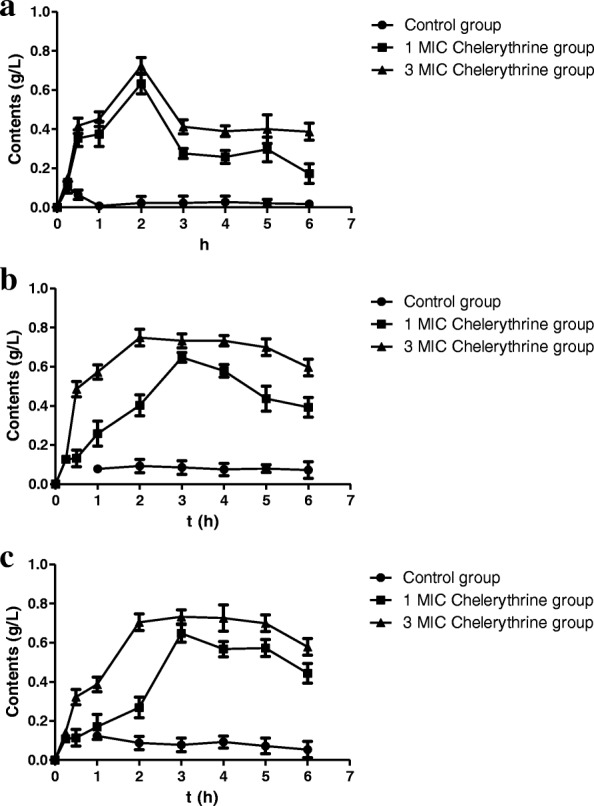
Changesinthe soluble protein concentration against SA (**a**), MRSA (**b**) and ESBLs-SA(**c**)

Fig. 3 showed that concentrations of soluble proteins were increased with increasing the concentrations of chelerythrine, demonstrating that chelerythrine may cause damage to bacteria and lead to exclusion of soluble proteins, which can, in turn, result in offsetting parts of the proteins consumed by bacteria. The concentrations reached a maximum for SA at 2 h and for MRSA and ESBLs-SA at 2.5 h.

#### Changes in levels of six bacterial proteins reavealed by SDS-PAGE analysis

**Fig. 4:**
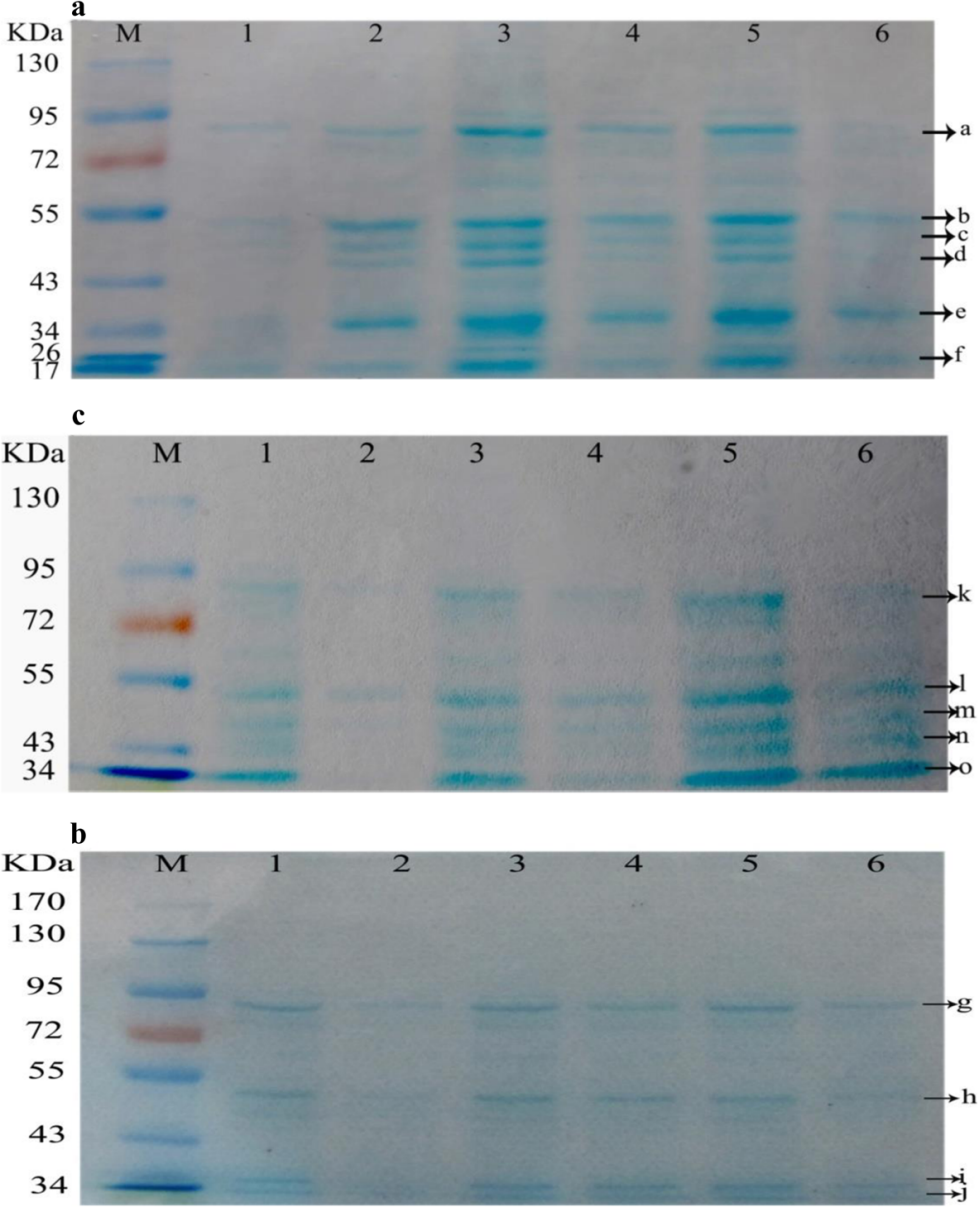
Changes in SDS-PAGE profiles of SA (**a**), MRSA (**b**) and ESBLs-SA (**c**) affected by chelerythrine. Lanes 1, 3 and 5: Normal electrophoretic bands of SA, MRSA, and ESBLs-SA for 3 h, 6 h, 9 h respectively(control groups); Lanes 2, 4, 6: Electrophoretic bands of SA, MRSA, and ESBLs-SA affected by chelerythrine treatment for 3, 6 and 9 h, respectively

In Fig. 4a, six bands (a, b, c, d, e, and f) in map of SA exhibited more obvious changes.

In Fig. 4b, the g-band (72KDa-95KDa) became shallower while h-band (43KDa-55KDa), i-band (34KDa-43KDa) and j-band (34KDa) disappeared at 3 h.

In Fig. 4c, k-band (72KDa-95KDa), l-band (43KDa-55KDa), m-band (43KDa-55KDa), *n*-band (43KDa) and o-band (34KDa) were becoming shallower or even disappeared as compared with those of the corresponding control groups at 3 h, 6 h and 9 h, respectively, suggesting that chelerythrine inhibits the expression of these proteins and synthesis of ESBLs-SA.

#### Ultrastructural changes

**Fig. 5:**
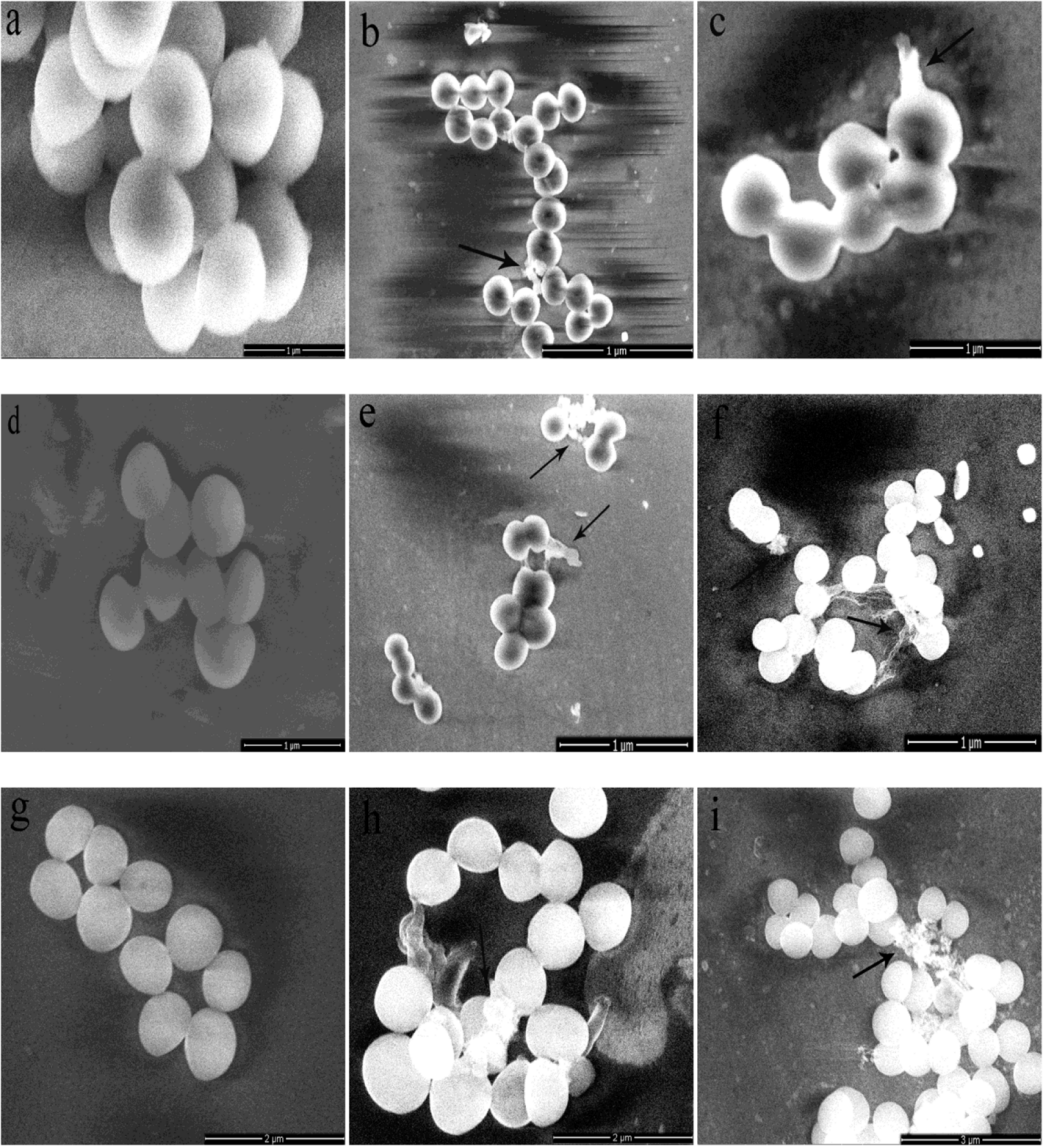
SEM images of SA (**a**, **b** and **c**), MRSA (**d**, **e** and **f**) and ESBLs-SA (**g**, **h** and **i**) treated by chelerythrine for 1.5 h (**b**, **e** and **h**), 6 h (**c**, **f**, **i**); and control (**a**, **d** and **g**)

The morphology of bacterial cells was examined by transmission electron microscopy (TEM) (Fig. 5). The bacteria of control group (Fig. 5a, d and g) displayed a spherical cell morphology that had a round and plump form without damaged surface. However, the external parts of the bacteria treated with chelerythrine adhered floc and a few cells became smaller or broken.

**Fig. 6:**
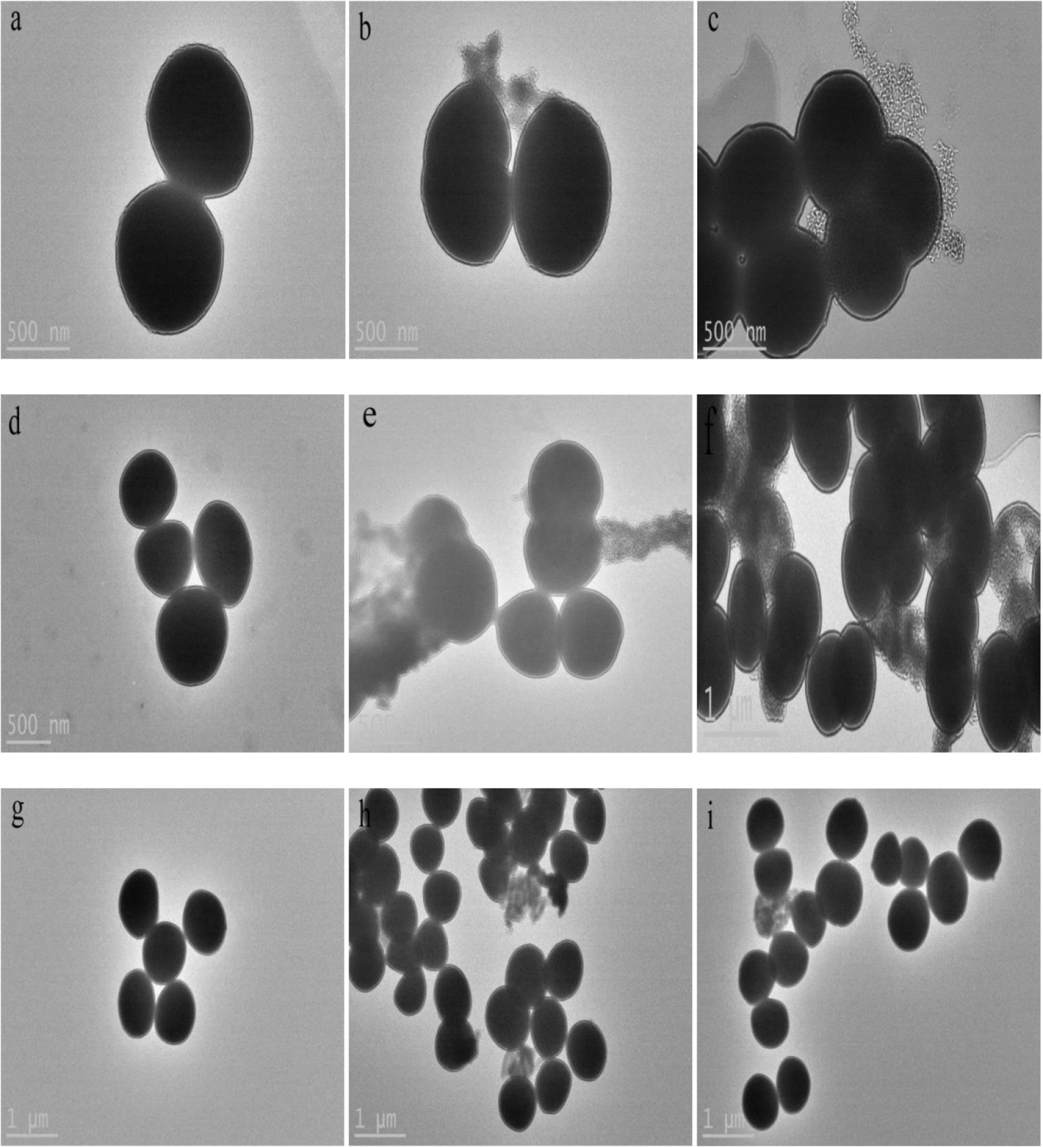
TEM images of SA (**a**, **b** and **c**), MRSA (**d**, **e** and **f**) and ESBLs-SA (**g**, **h** and **i**) treated by chelerythrine for 1.5 h (**b**, **e** and **h**), 6 h (**c**, **f** and **i**); and control (**a**, **d** and **g**)

In Fig. 6, TEM images showed that severe morphological changes were visualized in these chelerythrine-treated bacteria but such changes were not seen in control group (Fig. 6a, d and g), parts of the cell wall and cell membrane were damaged and some substances were leaked.

## Discussion

The present study showed that chelerythrine exhibited strong antibacterial effect. Results of HPLC suggested that chelerythrine in ethyl acetate fraction was highest and chelerythrine in petroleum ether fraction was lowest among four samples examined. However, the antibacterial activity of petroleum ether fraction was higher than that of ethyl acetate fraction, which might be due to the reason that other constituent of fractions also contribute to antibacterial functions [22] and the exact reasons need be further studied.

We valued the integrity of the bacterial cell wall by measuring contents of AKP. AKP in bacteria mainly exists between the cell wall and cell membrane of the bacteria. AKP will be leaked into the extracellular by increasing the permeability of bacterial cell wall. The results of AKP can reflect the integrity of the bacterial cell wall indirectly [23, 24]. The results in our study showed the permeability of cell walls of three bacterial strains could be increased by chelerythrine in a short- period of time.

Effects on cell membrane were also tested by measuring the leakage of intracellular soluble protein and the electrical conductivity of the bacteria solution [25]. We observed that chelerythrine imposed certain effects on the cell membrane of three strains of bacteria. There are positive correlations between protein leakage of proteins and chelerythrine concentration. It is suggested that the permeability of cell membrane of three bacteria was increased. 2.5 h later the bacterial protein concentrations were decreased gradually. This may be due to the reason that the effect of chelerythrine is decreased with time and the remaining bacteria still grow and consume the soluble proteins in the bacterial fluid. It also may be due to the reason that bacteria have been infringed and activated the self-healing mechanism, which directly caused a reduction of protein leakage. Therefore, the leaked proteins were not sufficient to offset the consumption of proteins.

SDS-PAGE electrophoresis bands protein were clearly changed when bacteria were treated with Chelerythrine (1 × MIC). In Fig. 4a, the protein bands of treatment groups were darker than the corresponding bands of control groups at 3 h because of the self-repairing mechanism of SA after being treated with chelerythrine and the synthesis of these proteins was accelerated to meet the needs of self-repairing cells. The six bands became shallower or even disappeared as compared with the corresponding bands of the control groups at 6 h and 9 h, which illustrated that there was certain suppression on normal metabolism of proteins. In brief, chelerythrine showed the inhibitory activity on SA by decreasing expression of the bacterial proteins. In Fig. 4b, the disappeared bands [h-band (43KDa-55KDa), i-band (34KDa-43KDa) and j-band (34KDa)] reappeared at 6 h and 9 h but were thinner than those of the control. Results showed that chelerythrine effectively inhibited the expression of such proteins in 3 h, the longer the MRSA were treated, the less effect the chelerythrine caused. On the other hand, the remaining MRSA continued to grow and the synthesis of these proteins was increased, indicating that chelerythrine can hinder protein expression of MRSA.

The morphology of bacterial cells was examined by transmission electron microscopy and results showed that parts but not all of cell membrane and cell wall of bacteria were broken, which this needs further explore.

## Conclusions

Chelerythrine is one of the major antibacterial ingredients in *T. asiatica*. The results obtained in this paper indicate that chelerythrine may be capable of destroying the cell wall and membrane of bacterial cells, leading to the increased membrane permeability, thereby causing the cellular contents to leak extracellularly. Meanwhile, chelerythrine could affect and hinder the bacterial protein expression and synthesis, eventually leading to cell death. Together, these results provide additional supporting evidence that chelerythrine can be a natural bactericide.

## Abbreviations

AKP: Alkaline phosphatase
HPLC: High Performance Liquid Chromatography
IC50: Half inhibitory concentration
IZ: Diameter of inhibition zone
K-B method: Disc diffusion method
MBC: Minimum bactericide concentration
MIC: Minimum inhibitory concentration
SEM: Scanning electron microscopy

## References

[1] Gibbons S (2004). Anti-staphylococcal plant natural products. Nat Prod Rep.

[2] Sharma A, Gupta S, Sarethy IP, Dang S, Gabrani R (2012). Green tea extract: possible mechanism and antibacterial activity on skin pathogens. Food Chem.

[3] Wright HT, Reynolds KA (2007). Antibacterial targets in fatty acid biosynthesis. Curr Opin Microb.

[4] Ramaraj T, Rajarajeswaran J, Murugaiyan P. Analysis of chemical composition and evaluation of antigenotoxic, cytotoxic and antioxidant activities of essential oil of *Toddalia asiatica* (L.) lam. Asian Pac J Trop Biomed. 2012;z3:1276–S1279. [View Article]

[5] Duraipandiyan V., Ignacimuthu S. (2009). Antibacterial and antifungal activity of Flindersine isolated from the traditional medicinal plant, Toddalia asiatica (L.) Lam. Journal of Ethnopharmacology.

[6] Karunai RM, Balachandran C, Duraipandiyan V, Agastian P, Ignacimuthu S. Antimicrobial activity of Ulopterol isolated from *Toddalia asiatica *(L.) lam.: a traditional medicinal plant. J Ethnopharmacol. 2012;140(1):161–5. [View Article] 10.1016/j.jep.2012.01.005 22265751

[7] Liu XC, Dong HW, Zhou L, Du SS, Liu ZL (2013). Essential oil composition and larvicidal activity of Toddalia asiatica roots against the mosquito *Aedes albopictus* (Diptera: Culicidae). Parasit Res.

[8] Santiagu SI, Christudas S, Veeramuthu D, Savarimuthu I. Antidiabetic and antioxidant activities of *Toddalia asiatica *(L.) Lam. leaves in Streptozotocin induced diabetic rats. J Ethnopharmacol. 2012;143(2):515–23. [View Article] 10.1016/j.jep.2012.07.006 22842651

[9] Kariuki HN, Kanui T, Yenesew A, Patel N, Mbugua PM. Antinocieptive and anti-inflammatory effects of *Toddalia asiatica* (L) Lam. (Rutaceae) root extract in Swiss albino mice. Pan Afr Med J. 2013;14:133. [View Article ] 10.11604/pamj.2013.14.133.2130 PMC3670198 23734278

[10] Shi L, Li D, Kang WY (2011). Research progress on chemical constituents and pharmacological activities of *Toddalia asiatica* (L.) lam. China Pharm..

[11] Wang F, Xu Y, Liu JK. New geranyloxy coumarins from* Toddalia asiatica*. J Asian Nat Prod Res. 2009;11(8):752–6. [View Article ] 10.1080/10286020903048975 20183319

[12] Muthumani P, Meera R, Devi P, Mohamed SA, Arabat S, Seshu Kumar Koduri LV, Manavarthi S. Chemical investigation of *Toddalia asiatica* Lin. and *Cardiospermum halicacabum* Lin. Int J Drug Formul Res. 2010;1:224–39. [View Ariticle]

[13] Shi L, Wang W, Ji ZQ, Kang WY (2014). Study on chemical constituents in methanol parts of *Toddalia asiatica*. China Pharm..

[14] Shi L, Ji ZQ, Zhang ZW, Li YY. Chemical constituents in ethyl acetate parts of *Toddalia asiatica* (Lin.) lam. China Pharm. 2013;16:1293–5.

[15] Chmura SJ, Dolan ME, Cha A, Mauceri HJ, Kufe DW, Weichselbaum RR (2000). In vitro and in vivo activity of protein kinase C inhibitor chelerythrine chloride induces tumorcell toxicity and growth delay in vivo. Clin Cancer Res.

[16] Zdařilová A, Malíková J, Dvořák Z, Ulrichováa J, Šimáne kV (2006). Quaternary isoquinoline alkaloids sanguinarine andchelerythrinein vitro and in vivo effects. Chem Listy.

[17] Niu XF, Zhou P, Li WF, Xu HB (2011). Effects of chelerythrine, a specific inhibitor of cyclooxygenase-2, on acute inflammation in mice. Fitoterapia.

[18] Miao F, Yang XJ, Ma YN, Zheng F, Song XP, Zhou L (2012). Structural modification of Sanguinarine and Chelerythrine and their in vitro Acaricidal activity against Psoroptescuniculi. Chem Pharm Bull.

[19] Szeto CC, Lai KB, Chow KM, CYK S, Wong YK, PKT L (2005). Differential effects of transforming growth factor-beta on the synthesis of connective tissue growth factor and vascular endothelial growth factor by peritoneal mesothelial cell. Nephron Exp Nephrol.

[20] Wang PQ, Yin ZH, Kang WY (2013). Research Progress on Pharmacological Activities of Chelerythrine. China. J Chin Mater Med.

[21] Wei JF, Zhang Q, Zhao L, Kang WY. Antimicrobial activity of *Musa basjoo *in vitro. Chin J Exp Med Formul. 2010;16(17):69–71. [View Article]

[22] Ding W, Wen CF, Chen JH, Huang KX, Dong AW (2007). Primary study on antibacterial activity of extracts from *Toddalia asiatica* (Linn.) lam. Biomass. Chemical. Engineering.

[23] Hara S, Yamakawa M (1995). Moricin, a novel type of antibacterial peptide isolated from the silkworm, *Bombyx mori*. J Biol Chem.

[24] Lan WQ, Xie J, Hou WF, Li DW. Antimicrobial Activity and mechanism of complex biological fresh-keeping agents against *Staphylococcus sciuri*. Nat Prod Res Dev. 2012;24:741–6. 753.[View Article]

[25] Liu SX, Wei HP, Ceng J, Yang JQ. Studies on antibacterial mechanism of the volatile oil from *Eupatorium adenophorum *antibacterial mechanism of *Staphylococcus aureus*. Chin J Hosp Pharm. 2012;32:1742–5. [View Article]

